# 1165. Seroprevalence of Chagas Disease among Latin American Children Living in New York

**DOI:** 10.1093/ofid/ofab466.1358

**Published:** 2021-12-04

**Authors:** Andrew S Handel, Harriet Hellman, Egar Flores, Christy Beneri

**Affiliations:** 1 Stony Brook Children’s Hospital, Stony Brook, NY; 2 Hampton Community Healthcare, Southampton, New York

## Abstract

**Background:**

Approximately 300,00 individuals in the United States are estimated to have Chagas disease. To date, only one seroprevalence study in the US has included children. Diagnosis during childhood prevents irreversible sequelae and is better tolerated than during adulthood. Seropositive children may be difficult to identify, as those infected vertically may have never visited an endemic region. We sought to identify children with Chagas disease through a pilot study of serology and risk factors.

**Methods:**

Participants were recruited from Stony Brook University Hospital (SBUH) or an ambulatory pediatric office, both in Suffolk County, New York (population: 1,476,000; 20.2% Hispanic or Latino). Study participants were 1 - 25 years old, resided in Suffolk County, and either the child and/or the child’s mother was born in or had long-term residence (≥ 3 years) in Latin America. *T. cruzi* serum IgG was determined with a Chagatest ELISA (Weiner Lab) or a Chagas Detect Plus Rapid Test (InBios). Positive screens were confirmed with a second serologic test at the CDC. Participants completed a survey of demographics and Chagas disease knowledge and risk factors, in English or Spanish. Descriptive statistics were applied. SBUH IRB provided study approval.

**Results:**

We enrolled 93 children (Table 1). Three (3.2%) had a positive IgG screen, of which only one had a confirmed infection (1.1%). This was a 17-year-old who had lived in a rural adobe home and moved to the US at 8 years old. No children or their mothers recalled being bitten by or seeing triatomine insects in their Latin American homes. Of 27 children whose mothers had been screened for infection, 13 were born to 3 mothers with confirmed Chagas disease; all 13 children were seronegative. Of 8 participants reporting other family members with Chagas disease, all were seronegative.

Demographics of 93 participants screened for Chagas disease

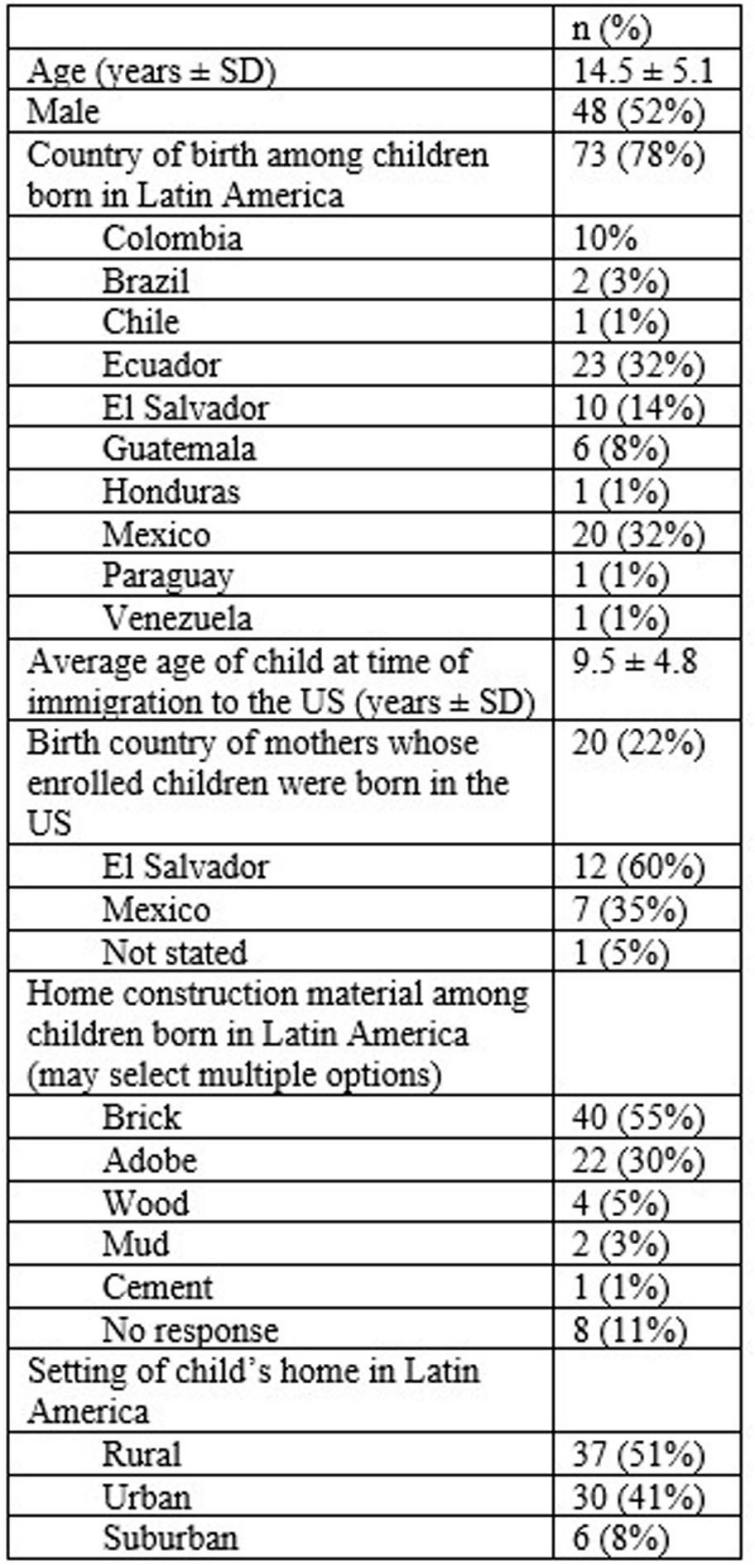

**SD:**

standard deviation; US: United States

**Conclusion:**

Without reliable tools for identifying those at greatest risk of Chagas disease, universal screening of children born in high-risk Latin American regions remains a reasonable strategy. In addition, screening mothers born in Latin America is likely a more cost-efficient means to evaluate second-generation children. A tremendous knowledge gap of pediatric Chagas disease in the US remains.

**Disclosures:**

**All Authors**: No reported disclosures

